# Neuron‐Derived Extracellular Vesicles as a Blood‐Based Biomarker for Alpha‐Synuclein Across Synucleinopathies

**DOI:** 10.1002/alz70856_106641

**Published:** 2026-01-07

**Authors:** Erez Eitan, Dina Ayupova, Olga Volpert, Alexander Gekas

**Affiliations:** ^1^ Neurodex, INC, Natick, MA, USA; ^2^ NeuroDex INC, Natick, MA, USA

## Abstract

**Background:**

Lewy body pathology, a hallmark of synucleinopathies, is present in most Parkinson's disease patients and approximately 30% of dementia cases, affecting over 24 million people worldwide. While cerebrospinal fluid (CSF) alpha‐synuclein (aSyn) seeding assays have emerged as promising diagnostic tools, they remain invasive and insufficient for widespread clinical implementation. The development of reliable blood‐based biomarkers is crucial for advancing early diagnosis and disease monitoring.

**Method:**

Plasma NDEs were isolated using ExoSORT, and aSyn was quantified using the Mesoscale kit and a custom‐developed Luminex assay.

**Result:**

Assay validation included specificity, precision, and compatibility testing. Key performance metrics included within‐batch variability (7%; 10 individuals, 3 repeats), between‐batch variability (13%; two pooled plasma samples, 17 batches), and assay linearity (*R* = 0.73, *p* < 0.01). The clinical potential of the assay was demonstrated across four independent cohorts:

**Cohort I:**

Healthy (*N* = 77, 19±21 pg/ml), synucleinopathy (*N* = 113, 217±401 pg/ml), and Alzheimer's Disease (*N* = 37, 235±598 pg/ml); AUC between synucleinopathy and healthy: 0.86 (*p* <0.001). **Cohort II**: Healthy (*N* = 9, 8±9 pg/ml) vs. Lewy Body Dementia (*N* = 37, 80±109 pg/ml); AUC: 0.91 (*p* <0.001). GBA mutation status did not affect aSyn levels. **Cohort III** (postmortem‐confirmed): Healthy (*N* = 15, 62±110 pg/ml), Alzheimer's without Lewy bodies (*N* = 15, 109±76 pg/ml), Lewy Body Dementia (*N* = 15, 178±92 pg/ml); AUC between LBD and healthy: 0.92 (*p* <0.001), and between AD and LBD: 0.76 (*p* <0.02). **Cohort IV**: Healthy (*N* = 15, 29±32 pg/ml) vs. Dementia (*N* = 45, 142±307 pg/ml). Tau pathology did not influence aSyn levels among dementia patients. We are now in the process of analyzing additional cohorts with over 900 subjects.

**Conclusion:**

NDE‐associated aSyn is significantly elevated in synucleinopathy patients across multiple cohorts, supporting its potential as a blood‐based biomarker. Further work is needed to standardize preanalytical conditions, establish a unified diagnostic threshold, and evaluate assay sensitivity at the individual level for clinical application.

